# Neffella xylocopae gen. nov., sp. nov., a novel host-specific gut symbiont of Xylocopa carpenter bees in the family Orbaceae

**DOI:** 10.1099/ijsem.0.007119

**Published:** 2026-07-14

**Authors:** Alan Emanuel Silva Cerqueira, Jo-anne C. Holley, Sarah C. Hatcher, Pedro Marcus Pereira Vidigal, Laila E. Phillips, Nancy A. Moran

**Affiliations:** 1 Department of Integrative Biology, The University of Texas at Austin, Austin, TX, USA; 2 Núcleo de Análise de Biomoléculas, Universidade Federal de Viçosa, Viçosa, Minas Gerais, Brazil

**Keywords:** bacteria, incipiently social bees, microbiome, Xylocopini, pangenomics, phylogenomics

## Abstract

Bee-associated *Orbaceae* species aid in the metabolism of plant polysaccharides, toxic sugars and urea and stimulate the immune system. In honeybees and other eusocial bees, microbial transmission occurs through hive contact and social interactions, favouring the emergence of host-specific strains. While most solitary bees acquire their microbiota from the environment, large carpenter bees (genus *Xylocopa*) exhibit facultative or incipient social behaviour that might enable direct transmission. This behaviour might have contributed to host specialization of *Xylocopa*-associated bacteria, such as the genus *Xylocopilactobacillus* and novel species belonging to the genus *Lactobacillus* and the family *Bifidobacteriaceae*. Evidence of an apparent *Xylocopa*-specific *Orbaceae* clade has also been observed. Here, we isolated and characterized AC157Xtp^T^, a novel strain in the family *Orbaceae*, from the gut of *Xylocopa tabaniformis parkinsoniae*. The optimal growth occurs anaerobically at 30–35°C, 0–0.5% salinity and pH 6–7. The predominant fatty acids were C_18:1_ *ω*6c and/or C_18:1_ *ω*7c (45.9%), followed by C_16:0_ (35.0%) and C_14:0_ (7.8%), consistent with those reported for members of the family *Orbaceae*. The cell size of AC157XtpT was ~0.5–1.6 µm in length and 0.4–0.7 µm in width, with coccoid to small rod-shaped morphology under scanning electron microscopy. The average nucleotide identity (ANI) and digital DNA–DNA hybridization (dDDH) scores between AC157Xtp^T^ and other *Orbaceae* ranged from 69.99 to 72.88% for ANIb, from 0.59 to 0.83 for TETRA values and from 20.1 to 26.7% for dDDH, measures far below species thresholds. Combined with the average amino acid identity (AAI) and percentage of conserved proteins (POCP) at the lower end of the *Orbaceae*-specific genus boundary range, and the phylogenomic tree placing AC157Xtp^T^ in a separate monophyletic clade sister to *Orbus* and *Frischella*, these data support the classification of AC157Xtp^T^ as a representative of a novel genus within the family *Orbaceae*. In conclusion, AC157XtpT (=NCIMB 15593T=ATCC TSD-486^T^) represents the type strain of *Neffella xylocopae* gen. nov., sp. nov. The GenBank accession numbers are CP133583 (genome) and PQ456091 (16S rRNA gene).

## Introduction

The eusocial corbiculate bees, including honeybees, bumblebees and stingless bees, usually harbour a dominant microbiota composed mainly of species of the families *Lactobacillaceae*, *Bifidobacteriaceae*, *Acetobacteraceae* and *Orbaceae* and the genus *Snodgrassella* [1,6]. These bacteria may benefit their hosts by contributing to digestion, immune stimulation, detoxification and protection against pathogens, with these functions mostly documented for honeybees [1]. These micro-organisms are transmitted each generation by contact with the hive environment [4,6, 7] and by social transmission from queens to offspring, as observed in bumblebees [7] and from older to younger workers [2,4, 6, 8, 9] via faecal–oral routes [4,7,10].

By enabling direct transmission within colonies, sociality has favoured the emergence of host-specific strains that have diversified with their hosts [1,4, 11] since the emergence of the corbiculate clade 80 million years ago [1,2]. These include members of the genera *Frischella* and *Gilliamella*, belonging to the insect-restricted family *Orbaceae* [1]. Based on studies in honeybees (*Apis mellifera*), these *Orbaceae* members have been implicated in the digestion of plant polysaccharides, metabolism of toxic sugars, utilization of host waste nitrogen, protection from pathogens or stimulation of the immune system [12,17].

In solitary bees, microbial acquisition occurs mainly from the environment [18,19], with flowers acting as hubs for interspecies microbial transmission, as shown for megachilid bees [18]. Host-specific strains co-diversifying via social transmission are generally absent, and the microbiota exhibit higher variability among individual hosts [19]. However, some carpenter bees (tribe Xylocopini) can be facultatively or incipiently social. These include the large carpenter bees (genus *Xylocopa*), which are widespread in the tropical and subtropical regions of most continents [20]. *Xylocopa* species can have social interactions between generations [21], potentially enabling social transmission from older to younger bees [21]. Such transmission might contribute to the evolution of *Xylocopa-*associated bacteria that co-evolved with these bees, such as the recently described genus *Xylocopilactobacillus* comprising two species (*Xylocopilactobacillus apicola* and *Xylocopilactobacillus apis*), and the novel species *Lactobacillus xylocopicola*, *Bombiscardovia nodaiensis*, *Bombiscardovia apis* [22], *Bifidobacterium xylocopae* and *Bifidobacterium aemilianum* [23]. Some *Xylocopa* species harbour a distinctive gut microbiota related to that of eusocial bees, including members of the families *Orbaceae*, *Lactobacillaceae* and *Bifidobacteriaceae*, suggesting that social transmission may help to maintain such associations [22,24,26].

Previous research showed that three different *Xylocopa* species collected near Austin, TX, USA (*Xylocopa tabaniformis parkinsoniae*, *Xylocopa micans* and *Xylocopa mexicanorum*) harbour *Orbaceae* species that are closely related to *Gilliamella* species from bumblebees . These bees also harboured strains forming a clade closely related to *Frischella perrara* from honeybees [26]. Closely related *Orbaceae* strains were also detected in *Xylocopa* species collected in CA and AZ [24]. Here, we observed the occurrence of a *Xylocopa-*specific *Orbaceae* clade and further isolated, from a *Xylocopa tabaniformis parkinsoniae* gut homogenate, a representative strain characterized as a novel species belonging to a novel *Xylocopa-*associated genus in the family *Orbaceae*.

## Bacterial isolation and identification

In order to isolate and describe a member of the *Xylocopa*-associated *Orbaceae* clade, we first collected *Xylocopa* species in Austin, TX (Table S1, available in the online Supplementary Material), and submitted DNA extracted from their gut homogenates for 16S rRNA amplicon sequencing (see the Supplementary Material for additional information). We obtained *Orbaceae* amplicon sequence variants (ASVs) from most of the *Xylocopa tabaniformis parkinsoniae* and *X. micans* samples, but not from the *Xylocopa virginica* samples (Fig. S1), as previously observed. A putative *Xylocopa*-specific clade was observed for *Orbaceae* ASVs when our sequences were analysed together with previous data [26] (Fig. S2).

The gut homogenates with a higher prevalence of *Orbaceae* (Fig. S1) were selected and used for serial dilution and isolation of bacteria. The selected stocked gut homogenates were thawed, 10 µl was diluted in Insectagro® DS2 and 40 µl from the 10^−2^ to 10^−5^ dilutions were plated in Petri dishes containing heart infusion agar supplemented with 5% v/v sheep’s blood medium (HIAb). We incubated the plates at 32°C in an anaerobic chamber until enough bacterial growth had been achieved (2–5 days); then, isolated colonies were passed to new plates. Pure colonies were collected from plates, added to 40 µl molecular-grade water and lysed by incubation for 10 min at 99°C in an Eppendorf thermocycler. The isolates whose lysates showed positive amplification with *Orbaceae*-specific primer pair 27F (AGAGTTTGATCMTGGCTCAG) and Orb742R (ATCTCAGCGTCAGTATCTGTCCAGAA) [27], targeting the region V1–V4 of the 16S rRNA gene, were grown on Insectagro® DS2 broth for 48 h and stocked in 20% v/v glycerol at −80°C.

The PCR products were sent for Sanger sequencing at the DNA Sequencing Facility at the Center for Biomedical Research Support at the University of Texas at Austin. The sequences were used as queries in blastn searches against the Nucleotide collection database of the NCBI blast server (https://blast.ncbi.nlm.nih.gov/). The isolate AC157Xtp^T^ originating from the gut of *Xylocopa tabaniformis parkinsoniae* Xtp_8 (Fig. S1, Table S1) aligned with members of the family *Orbaceae* and was selected for further study.

**Fig. 1.**
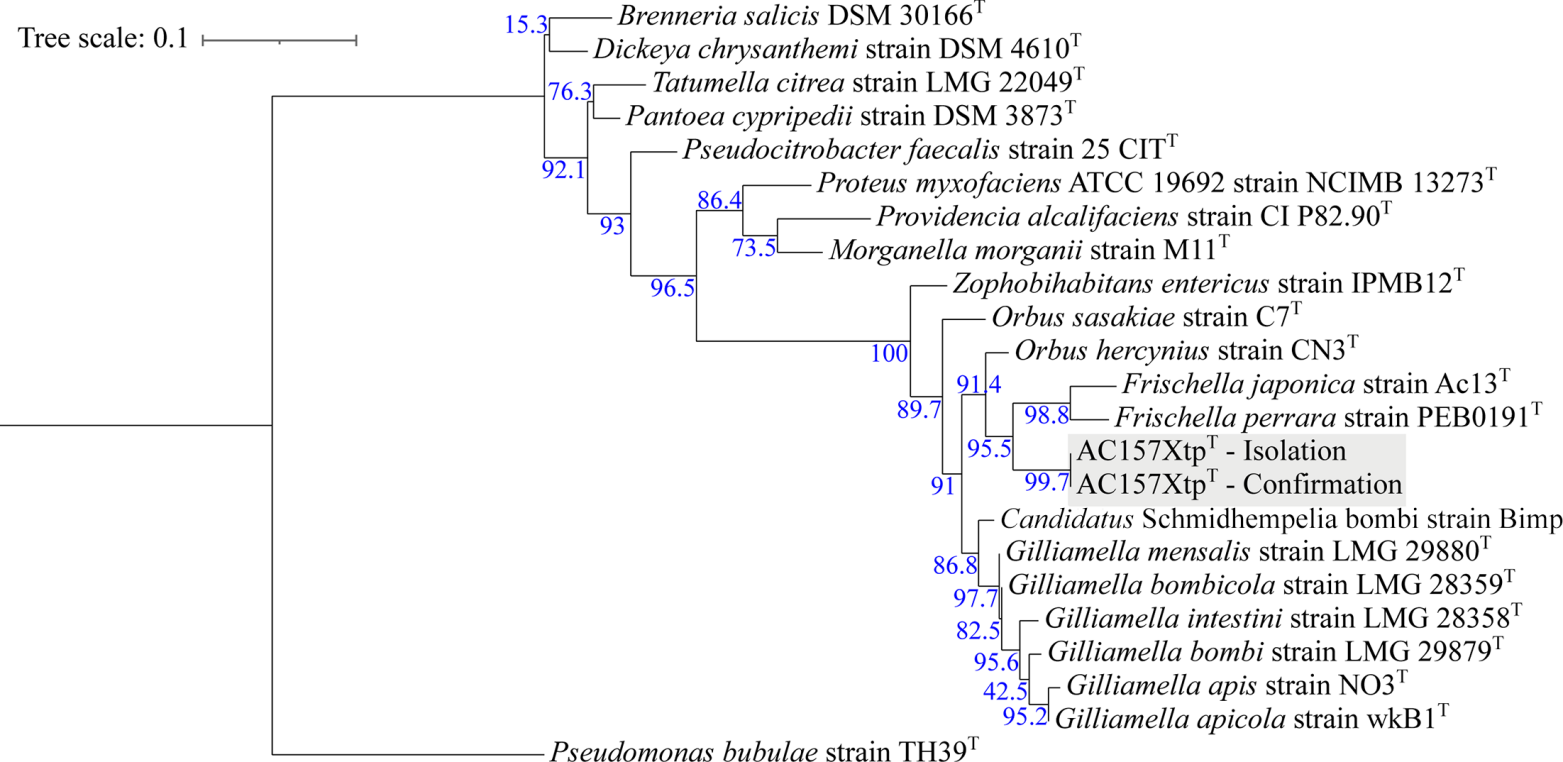
Maximum-likelihood phylogenetic inference based on the V1–V4 region of the 16S rRNA gene showing the placement of AC157Xtp^T^ in relation to type strains within the family *Orbaceae*. Values on the branches represent the bootstrap percentages based on 1,000 replicates. The superscript ‘T’ refers to the type strain. ‘AC157Xtp^T^ – Isolation’ refers to the first round of sequencing used for the identification of the AC157Xtp^T^ isolate; ‘AC157Xtp^T^ – Confirmation’ refers to the second round of sequencing for confirming the purity of the AC157Xtp^T^ prior to genome sequencing. The tree was rooted with *Pseudomonas bubulae* strain TH39. The scale bar represents 0.1 nucleotide substitutions per position. Accession numbers for the sequences used are available in Table S2.

We downloaded closely related reference sequences and aligned them with the V1–V4 region of the 16S rRNA gene of AC157Xtp^T^ using MAFFT and then estimated phylogenetic relationships using a maximum-likelihood method with 1,000 ultrafast bootstrap replicates in IQ-TREE [28]. The TPM3+I+G4 nucleotide substitution model was selected according to the Bayesian information criterion (BIC) by ModelFinder [29] implemented in IQ-TREE. This analysis placed AC157Xtp^T^ within the family *Orbaceae*, close to *Frischella* but forming a putative *Xylocopa*-specific clade (Fig. 1), similarly to the clade observed for the *Xylocopa*-associated *Orbaceae* ASVs (Fig. S2). A second round of sequencing retrieved an identical sequence, confirming the purity of AC157Xtp^T^ stocks (Fig. 1).

## Genomic features

### Genome sequencing

The AC157Xtp^T^ strain was sent for genome sequencing at the Microbial Genome Sequencing Center (MIGS, currently SeqCenter, Pittsburgh, PA, USA), using a combination of Illumina Shotgun short-read sequencing and Oxford Nanopore Technology (ONT) long-read sequencing.

**Fig. 2.**
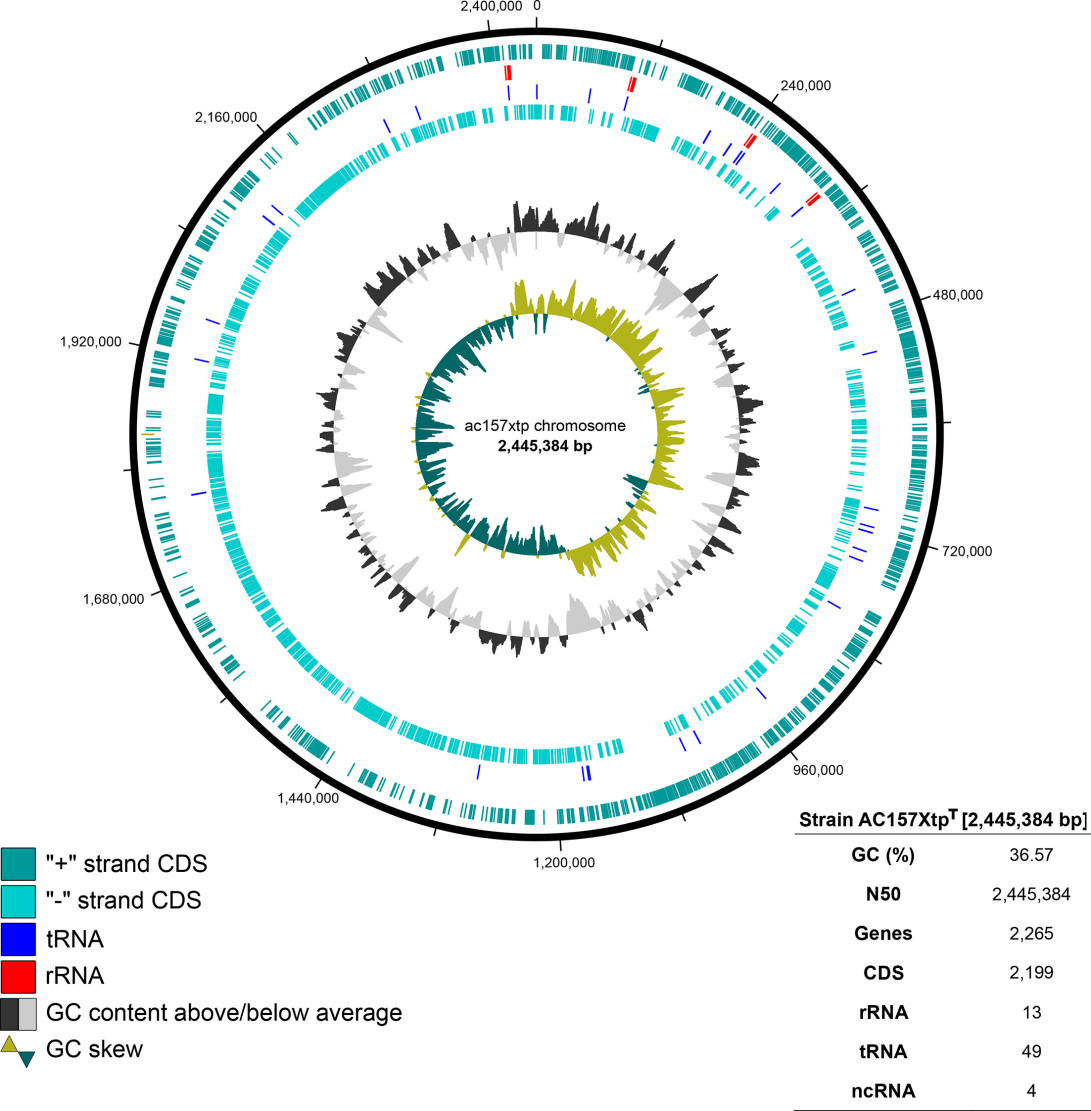
Visualization of the annotated circular genome of AC157Xtp^T^ and its genomic features.

The pipelines bcl2fasta (available at https://support.illumina.com/sequencing/sequencing_software/bcl2fastq-conversion-software.html) and Porechop (available at https://github.com/rrwick/Porechop) were used for quality control and data trimming of Illumina and ONT sequencing, respectively. Illumina and ONT reads were hybrid-assembled into a single contig using Unicycler [30] by MIGS, and assembly statistics were recorded with QUAST [13]. The genome was assembled into a single contig of 2,445,384 bp (~2.45 MB) and a G+C DNA content of 36.57 mol%. The sequence was submitted for annotation with the NCBI Prokaryotic Genome Annotation Pipeline (PGAP) [31,33], and the visualization of the annotated circular genome and its features was generated with DNAPlotter [34]. A total of 2,265 genes were detected, of which 2,199 are coding DNA sequences (CDSs) and 66 are RNA genes – 13 rRNA, 49 tRNA and 4 ncRNA (Fig. 2).

### Checking genome purity

**Fig. 3.**
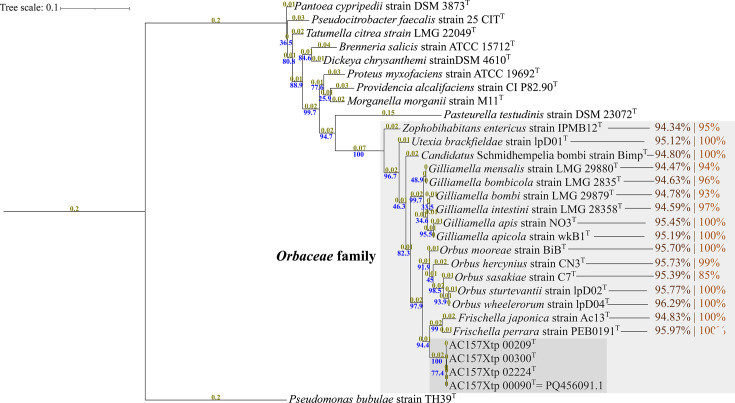
Maximum-likelihood phylogenetic inference showing the placement of each of the four full-length 16S rRNA gene copies retrieved from the AC157Xtp^T^ genome in relation to type strains within the family *Orbaceae*. Blue values on the branch represent the bootstrap percentages based on 1,000 replicates. The superscript ‘T’ refers to the type strain. Tree was rooted with *Pseudomonas bubulae* strain TH39. The scale bar represents 0.1 nucleotide substitutions per position, and olive values indicate branch lengths. The per cent identity and coverage values calculated by blastn between the query sequence (AC157Xtp^T^=PQ456091.1) and representative members of the family *Orbaceae* are shown in dark brown (left column) and light brown (right column), respectively. The accession numbers for the sequences used are available in Table S2.

Four copies of the full-length 16S rRNA gene were retrieved from the annotated genome. We calculated the per cent identity and coverage values between one copy of the 16S rRNA gene of strain AC157Xtp^T^ (gene locus AC157Xtp_00090, NCBI accession PQ456091.1) and the reference sequences of other *Orbaceae* species (Table S2) using blastn at the NCBI website. The four copies were used for phylogenetic inference to confirm the purity and consistency of the assembled genome. Phylogenetic inference was performed as previously described, using the BIC-selected TIM3+F+I+R2 nucleotide substitution model. The resulting tree topology confirms the placement of AC157Xtp^T^ within the family *Orbaceae*, forming a putative *Xylocopa*-specific lineage sister to the *Frischella* clade (Fig. 3). The 16S rRNA sequence of AC157Xtp^T^ shares identities ranging from 94.34 to 96.29% with other *Orbaceae* genera. These values are below the species-level boundary of 98.65% [35]. The general genus boundary of 94.5–95% similarity [36] seems not to accommodate *Orbacea*e species, since the identities between AC157Xtp^T^ and species of the phylogenetically distant genera *Gilliamella* and *Utexia* are >95%, whereas the identity between AC157Xtp^T^ and the more closely related *Frischella japonica* is lower, ~94.83% (Fig. 3). Indeed, the evolutionary dynamics of the 16S rRNA gene can be genus-specific, causing 16S rRNA phylogenies to differ from core genome phylogenies [37,38].

### Pangenomics and phylogenomics

The AC157Xtp^T^ and available *Orbaceae* annotated genomes were submitted to the JSpeciesWS web server [39] to calculate the average nucleotide identity (ANI) based on blast+ (ANIb) [40], the ANI based on MUMmer (ANIm; data not shown) [41] and the tetranucleotide-derived z-score correlations (TETRA) [42] using the Tetra Correlation Search (TCS) feature. TETRA values >0.99 between a query and a reference genome generally support the species circumscription of ANI values >95%, indicating that the genomes belong to the same species [43]. We also calculated the digital DNA–DNA hybridization (dDDH) values using the Genome-to-Genome Distance Calculator (GGDC) 3.0 [44], which is an *in silico* alternative to wet-lab DNA–DNA hybridization (DDH), with lower variability and which assumes that genomes belong to the same species if dDDH values are above the 70% DDH threshold [45]. Additionally, we calculated the average amino acid identity (AAI) using the software EzAAI [46], which uses Prodigal [47] to predict and extract CDS profiles from the genomes and MMSeqs2 [48] to perform pairwise AAI calculations on these profiles. AAI values of 95–96% are commonly used to delimit species [49,50], and values of 60–80% are used to delimit different genera [50]. The AAI between available *Orbaceae* genomes indicates that different genera range from 68.89% (between *Candidatus* Schmidhempelia bombi and *Orbus sturtevantii*) to 76.07% (between *Orbus sasakiae* and *F. perrara*) (Table S3). Pairwise comparisons between AC157Xtp^T^ and the *Orbaceae* genomes showed values between 69.99 and 72.88% for ANIb, 0.59 and 0.83 for TETRA, 20.10 and 26.70% for dDDH and 69.07 and 74.82% for AAI (Tables 1 and S3). All these measures are far below the 95%, 0.99, 70% and 95% species thresholds for ANI, TETRA, dDDH and AAI, respectively, and the AAI values are within the range of genus delimitations.

**Table 1.** ANIb, TETRA, dDDH, AAI and POCP calculated between AC157Xtp^T^ and other *Orbaceae* reference genomes

Reference	AC157Xtp^T^ (2445384 bp)	Genome size	G+C content	ANIb	TETRA	dDDH	AAI	POCP matrix	POCP nf
Genome ID	Species	Mbp	mol%	(%)	values	(%)	(%)	(%)	(%)
GCF_000471645.3	*Candidatus* Schmidhempelia bombi strain Bimp	2.2	36.0	70.73	0.77	26.7	69.07	60.3	59.9
GCF_014489845.1	*Frischella japonica* strain Ac13^T^	2.7	34.5	72.31	0.78	21	73.02	58.2	58.3
GCF_000807275.1	*Frischella perrara* strain PEB0191^T^	2.7	34.0	72.13	0.79	22.1	73.05	59.2	59.0
GCF_000599985.1	*Gilliamella apicola* strain wkB1^T^	3.1	33.5	72.19	0.81	22.5	72.08	55.2	54.7
GCF_001690755.1	*Gilliamella apis* strain P62G	2.5	34.5	72.34	0.74	22.3	72.22	59.8	60.2
GCF_900103085.1	*Gilliamella mensalis* strain LMG 29880^T^	2.3	35.5	72.07	0.81	21.7	72.09	61.4	61.4
GCF_900094945.1	*Gilliamella bombicola* strain R-53248^T^	2.3	36.0	72.17	0.83	21.6	72.28	61.0	60.7
GCF_900103255.1	*Gilliamella bombi* strain LMG 29879^T^	2.6	34.5	72.32	0.83	21.1	72.16	59.5	59.8
GCF_900094935.1	*Gilliamella intestini* strain LMG 28358^T^	2.5	34.5	72.15	0.82	20.1	71.98	59.5	59.8
GCF_011745665.1	*Zophobihabitans entericus* IPMB12^T^	2.7	39.5	70.34	0.60	23.3	70.59	57.5	57.8
GCF_036251705.1	*Utexia brackfieldae* strain lpD01^T^	2.4	41.5	69.99	0.59	23.9	69.26	57.5	57.8
GCF_039542345.1	*Orbus sasakiae* strain JCM 18050^T^	2.9	37.5	72.5	0.74	20.2	74.82	61.8	61.8
GCF_036251935.1	*Orbus wheelerorum* strain lpD04^T^	2.9	36.0	72.49	0.74	20.9	74.10	59.1	59.5
GCF_036251875.1	*Orbus sturtevantii* strain lpD02^T^	2.9	37.0	72.16	0.76	20.3	73.61	58.9	58.1
GCF_003634275.1	*Orbus hercynius* strain CN3^T^	2.4	39.0	72.07	0.70	20.6	73.77	62.0	61.9
GCF_036251205.1	*Orbus mooreae* strain BiB^T^	2.9	35.5	72.88	0.74	20.4	74.31	58.1	57.1

To further confirm that AC157Xtp^T^ is different enough from other *Orbaceae* to be considered a novel genus, we performed the computation of the percentage of conserved proteins (POCP) with default parameters [51]. We employed two methods for pairwise all-vs-all protein alignment calculations with blastp: the POCP-matrix.py script (https://github.com/SilentGene/Bio-py/tree/master/POCP-calculator), which used PGAP-annotated *Orbaceae* and AC157Xtp^T^ protein files (.faa), and the blastp program from the blast+ package [52] for alignment calculations; and the POCP-nf workflow (version 2.3.6, https://github.com/hoelzer/pocp) [53,54], in which genomes in FASTA format were first annotated with the built-in Prokka [54], and alignments were calculated with the blastp mode of DIAMOND [55]. Both approaches returned POCP values higher than the 50% genus-level proposed threshold [51] for all pairwise comparisons within the *Orbaceae* family, indicating that this cutoff is not suitable for separating *Orbaceae* genera. The 50% POCP threshold is not universally applicable across bacterial families, and family-specific thresholds provide better resolution when the standard 50% cutoff lacks discriminative power [51,56]. Therefore, a suitable genus boundary range for members of *Orbaceae* might lie between 54.7 and 71.3%, and the POCP values obtained in pairwise comparisons between AC157Xtp^T^ and other *Orbaceae* genera were consistently at the lower end of this range (~54.7–62%) (Tables 1, S12 and S13), reinforcing that it is sufficiently different from other genera within the family. These results further support the classification of AC157Xtp^T^ as a novel species within a novel genus in the family *Orbaceae* (Table 1).

**Fig. 4.**
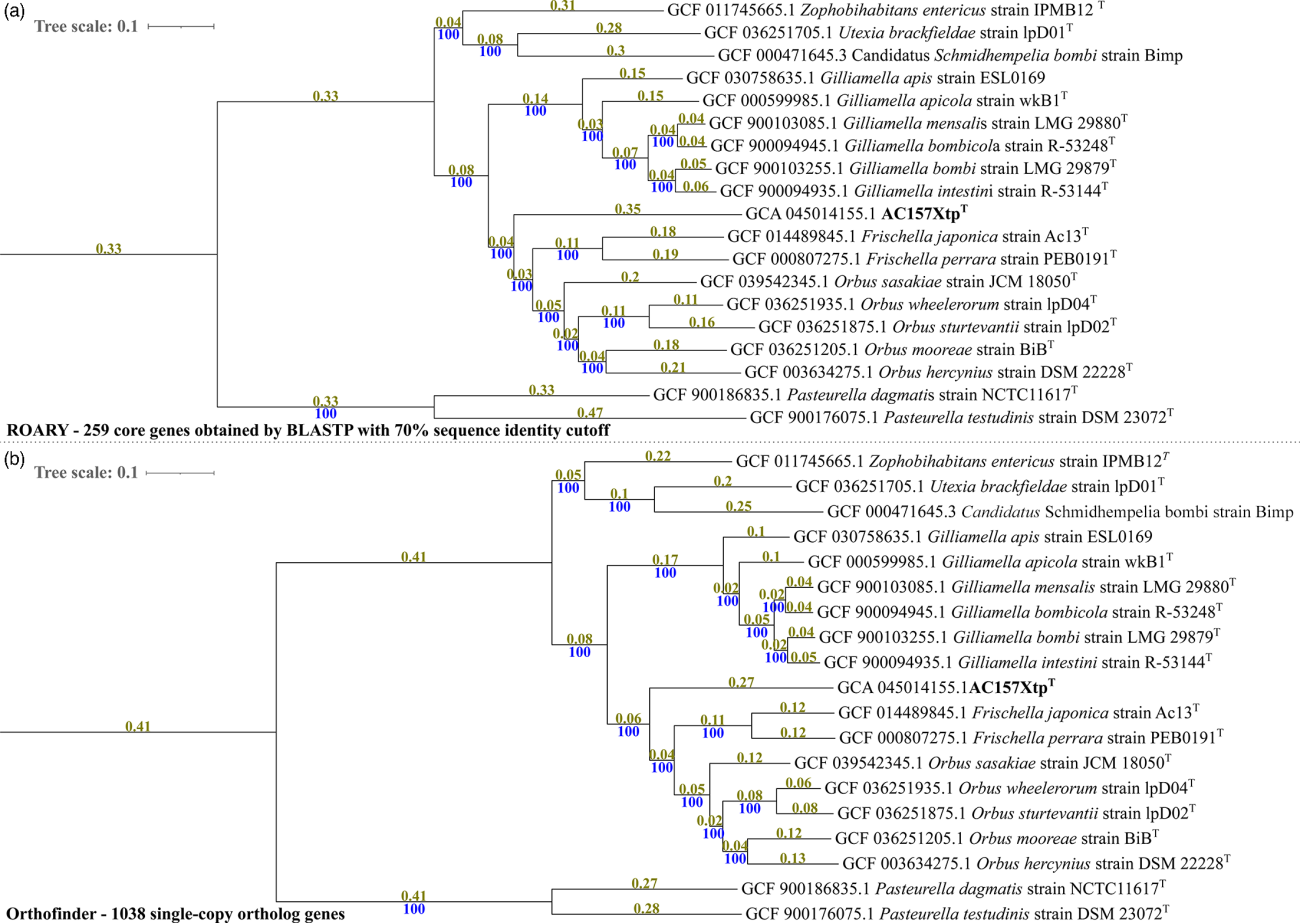
Maximum-likelihood phylogenetic tree showing the placement of the isolate AC157Xtp^T^ in relation to strains within the family *Orbaceae*. (**a**) The tree was constructed using the concatenated alignment of 259 core genes obtained by blastp with 70% sequence identity cutoff. (**b**) The same topology was obtained using the concatenated alignment of 1,038 single-copy orthologue genes. The blue values on branches represent the bootstrap percentage based on 1,000 replicates. The superscript ‘T’ refers to the type strain. The tree was rooted with the clade composed of *P. dagmatis* strain NCTC 11617^T^ and *P. testudinis* DSM 23072^T^ outgroups. The scale bar represents 0.1 nucleotide substitutions per position and olive values indicate branch lengths.

These *Orbaceae* genomes and the outgroups *Pasteurella testudinis* DSM 23072^T^ and *Pasteurella dagmatis* strain NCTC11617^T^ were submitted to pangenome determination with ROARY [57]. Coding regions of annotated genomes were selected, converted to protein sequences and pre-clustered. Sequences were submitted to blastp with a 70% sequence identity cutoff, clustered and checked for homology. Homologous groups containing paralogues were split into groups of true orthologues, and 259 core genes were detected, aligned by MAFFT [58], implemented in ROARY, and concatenated. Alignment gaps were trimmed with trimAl using the *automated1* option [59], and we made the maximum-likelihood phylogenetic inference with 1,000 bootstrap replicates with IQ-TREE [28] using the GTR+F+I+R5 nucleotide substitution model selected by BIC using ModelFinder [29] implemented in IQ-TREE. Additionally, 1,038 single-copy orthologue genes from the genomes were retrieved, aligned with MAFFT, concatenated and had their gaps trimmed with default configurations of OrthoFinder [60]. This trimmed concatenated alignment was used for maximum-likelihood phylogenetic inference using IQ-TREE with 1,000 bootstrap replicates and the BIC-selected model LG+F+I+G4, obtained by ModelFinder as described above. Both trees had the same topology, confirming the well-supported placement of AC157Xtp^T^ with 100% bootstrap within the *Orbaceae* family in a separate branch of a clade composed of the genera *Frischella* and *Orbus*. In addition, the branch lengths separating AC157Xtp^T^ from members of the genera *Frischella* (0.67–0.68/0.54) and *Orbus* (0.63–0.72/0.48–0.55), its closest relatives, can be higher than those separating *Utexia brackfieldae* and *Candidatus* Schmidhempelia bombi (0.58/0.37) and *U. brackfieldae* and *Zophobihabitans entericus* (0.67/0.52) (Fig. 4a, b). The tree topology indicates a separate evolutionary lineage, which, along with the branch lengths, confirms that it is different enough to be considered a separate genus. Taken together, all the presented results on pangenomic and phylogenomic analyses indicate that AC157Xtp^T^ is a novel species and should be considered a representative of a novel genus in the family *Orbaceae* (Table 1, Fig. 4).

### Metapangenomics: functional analysis

To explore the functional similarities and differences among AC157Xtp^T^ and other *Orbaceae* strains, the genomes were submitted to the EggNOG-mapper. This tool performs gene functional annotation via pre-computed orthology assignments of the eggNOG database of orthologue groups [61,62]. The bacterial genes were classified into 25 functional categories (Table 2). The AC157Xtp^T^ strain had the highest number of genes in the cell cycle control, cell division and chromosome partitioning (D) and translation, ribosomal structure and biogenesis (J) categories (Table 2, underlined) and the lowest in the cell wall/membrane/envelope biogenesis (M); post-translational modification, protein turnover and chaperones (O); amino acid transport and metabolism (E); nucleotide transport and metabolism (F); and coenzyme transport and metabolism (H) categories (Table 2, red).

**Table 2.** Comparison of the number of genes present in the 25 eggNOG functional categories between AC157Xtp^T^ and other *Orbaceae* strains Strain abbreviations: Nx, *Neffella xylocopae* AC157Xtp^T^; Oh, *Orbus hercynius* CN3^T^; Fp, *F. perrara* PEB0191^T^; Fj, *Frischella japonica* Ac13^T^; Ga, *Gilliamella apis* P62G; Gapc, *Gilliamella apicola* wkB1^T^; Gm, *Gilliamella mensalis* LMG 29880^T^; Gbcl, *Gilliamella bombicola* LMG 28359^T^; Gb, *Gilliamella bombi* strain LMG 29879^T^; Gint, *Gilliamella intestini* strain LMG 28358^T^; Ze, *Z. entericus* IPMB12^T^; Csb, *Candidatus* Schmidhempelia bombi.

COG_category*	Nx†	*Oh*	*Fp*	*Fj*	*Ga*	*Gapc*	*Gm*	*Gbcl*	*Gb*	*Gint*	*Ze*	*Csb*
Cellular processes and signalling												
[D] Cell cycle control, cell division, chromosome partitioning	45	36	38	36	35	40	38	36	35	36	39	42
[M] Cell wall/membrane/envelope biogenesis	**124**	142	147	152	137	167	137	133	140	168	156	147
[N] Cell motility	12	15	53	47	43	46	46	43	49	44	10	15
[O] Post-translational modification, protein turnover and chaperones	**60**	70	68	73	72	78	68	63	74	73	72	75
[T] Signal transduction mechanisms	41	49	63	62	48	63	42	46	38	40	57	38
[U] Intracellular trafficking, secretion and vesicular transport	58	69	63	69	51	63	57	50	59	66	84	68
[V] Defence mechanisms	33	27	45	44	42	44	33	41	38	44	31	30
[W] Extracellular structures	0	0	0	0	0	0	0	0	0	0	0	0
[Y] Nuclear structure	0	0	0	0	0	0	0	0	0	0	0	0
[Z] Cytoskeleton	0	0	1	0	0	1	1	0	0	1	2	2
Information storage and processing												
[A] RNA processing and modification	0	1	1	1	0	0	0	0	0	0	1	3
[B] Chromatin structure and dynamics	0	0	0	0	0	0	0	0	0	0	2	3
[J] Translation, ribosomal structure and biogenesis	185	183	184	184	183	179	178	178	182	180	183	181
[K] Transcription	129	132	138	136	160	198	135	140	126	129	179	86
[L] Replication, recombination and repair	130	119	120	135	116	178	147	131	185	207	127	119
Metabolism												
[C] Energy production and conversion	87	99	75	77	87	106	81	82	79	81	110	67
[E] Amino acid transport and metabolism	**121**	185	161	165	184	205	166	167	161	155	177	133
[F] Nucleotide transport and metabolism	**72**	85	90	84	91	97	87	89	87	85	90	75
[G] Carbohydrate transport and metabolism	111	135	126	165	216	260	153	167	115	136	209	92
[H] Coenzyme transport and metabolism	**86**	116	101	98	105	116	104	102	104	102	114	98
[I] Lipid transport and metabolism	46	47	73	62	57	59	42	51	52	44	60	39
[P] Inorganic ion transport and metabolism	107	147	140	129	150	166	124	130	121	117	137	101
[Q] Secondary metabolites biosynthesis, transport and catabolism	16	20	36	25	23	26	17	16	26	18	25	28
Poorly characterized												
[R] General function prediction only	0	0	0	0	0	0	0	0	0	0	0	0
[S] Function unknown	322	322	357	333	328	446	340	288	443	370	356	306

*Some genes belong to more than one COG category.

†Categories in which the AC157XtpT strain has the highest/lowest number of genes are underlined or bolded, respectively.

We also submitted PGAP-annotated AC157Xtp^T^ and other *Orbaceae* genomes to the Rapid Annotations using Subsystems Technology (RAST) server [63,64], using the Classic RAST option and preserving the original annotation (preserving gene calls). We obtained the classification of the annotated genes in the different functional SEED subsystem categories, representing collections of protein families with related functions. We compared this classification between AC157Xtp^T^ and some strains of other *Orbaceae* genera: *Orbus hercynius* CN3^T^, *Frischella perrara* PEB0191^T^, *Gilliamella apicola* wkB1^T^, *Zophobihabitans entericus* IPMB12^T^, and *Candidatus* Schmidhempelia bombi (Table S4). In this comparison, the proteins found exclusively in AC157Xtp^T^ strain are associated with the following subsystems: threonine degradation (l-threonine transporter protein and threonine dehydratase), branched-chain amino acid biosynthesis and glycine and serine utilization (threonine dehydratase), persister cells (HipA and HipB proteins), phosphoglycerate transport system (protein PgtP), toxin–antitoxin replicon stabilization systems (HigB toxin protein, HigA protein – antitoxin to HigB, RelE/StbE replicon stabilization toxin, RelB/StbD replicon stabilization protein – antitoxin to RelE/StbE, YafQ toxin protein and YoeB toxin protein), orphan regulatory proteins (sensory histidine kinase AtoS) and group II intron-associated genes (retron-type RNA-directed DNA polymerase). A putative deoxyribonuclease similar to the YcfH type, associated with DNA metabolism, was also only present in AC157Xtp^T^ (Table S4). The protein 3-methyl-2-oxobutanoate hydroxymethyltransferase, belonging to subsystems related to coenzyme A biosynthesis, and pantoate–beta-alanine ligase that also belongs to the folate biosynthesis cluster subsystem, were only found in the bee-associated AC157Xtp^T^, *Frischella* and *Gilliamella* strains (Table S4).

## Physiologic, morphologic and biochemical features

### Growth conditions

Overall, the AC157Xtp^T^ strain had better growth in anaerobic conditions compared to 5% CO_2_ incubation conditions. Minimal to no growth was observed under ambient atmospheric conditions. The AC157Xtp^T^ strain was tested for growth in agar media by inoculating 48-h-old colonies cultured in HIAb in four different 5% v/v sheep blood-supplemented media:Columbia blood agar (CBAb), trypticase soy agar (TSAb), heart infusion agar (HIAb), and Mueller–Hinton agar (MHAb). In addition, we also tested seven non-blood supplemented media, the lysogeny broth agar – Lennox modification (0.5% w/v NaCl), Mueller–Hinton agar (MHA), heart infusion agar (HIA), trypticase soy agar (TSA), 2X YTGA (2X yeast extract tryptone agar medium with 0.3% w/v glucose added), Columbia blood agar (CBA), and Lactobacilli de Man, Rogosa and Sharpe (MRS) agar (see Table S5 for media details). The plates were incubated anaerobically at 35°C for 48 h. Good growth with visible single colonies was observed in all the blood-supplemented media, in 2X YTGA and HIA. No signs of haemolysis were observed in the blood-supplemented media.

Then, we tested the capability of AC157Xtp^T^ to grow in different liquid media by inoculating 10 µl of 48-h-old Insectagro® DS2 cultures adjusted to OD_600_=0.1 or 10 µl of OD_600_=0.1 suspensions in 0.85% w/v NaCl prepared using colonies harvested from 48-h-old HIAb plates into 96-well plates containing 190 µl of Columbia broth (CB), trypticase soy broth (TSB), fastidious anaerobic broth (FAB), R-2A broth, brain heart infusion (BHI), Insectagro® DS2, SFM4Insect™ (with l-glutamine and sodium bicarbonate), 2X yeast extract tryptone broth+0.3% w/v dextrose/d-glucose (2X YTG), a modified version of heart infusion broth (HIB) and Lactobacilli MRS broth (see Tables S4 and S5 for media details). The plates were incubated for 72 h in anaerobic conditions at 35°C. The cultures had better growth according to OD_600_ measurements in CB, TSB, 2X YTG, Insectagro® DS2 and SFM4Insect™^T^ (Table S6). For further temperature, salinity and pH range experiments, liquid cultures were grown in Insectagro®, SFM4Insect™ or 2X YTG media for 24–48 h and adjusted to an OD_600_ of at least 0.5±0.1, instead of 0.1, to increase the starting number of cells and yield higher OD_600_ measures for the assays. Then, 10 µl of culture was inoculated into 96-well plates containing 190 µl of the appropriate medium in each well for 72 h.

The growth temperature range was assessed by inoculating 10 µl of 48-h-old Insectagro® DS2 and SFM4Insect™ cultures adjusted to OD_600_ of 0.5±0.1 in 96-well plates with 190 µl of Insectagro® and SFM4Insect™ in each well. The plates were incubated anaerobically at 4, 15, 20, 25, 30, 35, 37 and 40°C. The growth according to OD_600_ was measured at 0, 24, 48 and 72 h time points. Very little growth was observed at 4°C, and delayed growth was obtained at 15 and 20°C. For each medium, the highest OD_600_ was obtained at 24 h in the 25–40°C range, with better growth observed at 30–35°C (Table S7). Therefore, we conclude that the AC157Xtp^T^ strain has an optimum growth temperature between 30 and 35°C.

The salinity growth range was determined by inoculating 10 µl of 24-h-old 2X YTG and SFM4Insect™ cultures adjusted to an OD_600_ of 0.5±0.1 and 0.8±0.1, respectively, in 96-well plates with 190 µl of 2X YTG containing 0 to 3.5% w/v NaCl in 0.5% increments. The plates were incubated anaerobically for 72 h at 35°C. Growth was measured at 24, 48 and 72 h time points. The AC157Xtp^T^ strain can tolerate salinities up to 1.5% w/v with better growth up to 1.0% w/v. Faster growth was observed at 0% w/v salinity, reaching its peak OD_600_ at 24 h. Delayed growth was observed from 0.5 to 1.5% w/v salinity, with the highest average OD_600_ obtained at 0.5% w/v at 48 h. When the starting culture was increased to an OD_600_ of 0.8, better growth was also observed for the 0–1.0% w/v salinity range. The highest growth was also observed at 0.5% w/v but at 24 h instead of 48 h. Therefore, the optimum growth of AC157Xtp^T^ can be obtained at 0-0.5% w/v salinity (Table S8).

For the pH growth range determination, 10 µl of 48-h-old 2X YTG and SFM4Insect™ cultures were adjusted to an OD_600_ of 0.5±0.1 and inoculated in wells containing 190 µl of 12 different 2X YTG formulations ranging from pH 4 to 9.5 (0.5 pH increments). The plates were then incubated anaerobically for 72 h at 35°C. The AC157Xtp^T^ strain can tolerate a pH between 5 and 7.5. Very weak and delayed growth was observed at pH 5. Cultures at pH 5.5 and 7.5 can reach OD_600_ >0.1 only at 72 and 48 h, respectively, but have weak growth at 24 h. Cultures at pH 6, 6.5 and 7 had the highest growth at 24 and 48 h. The highest overall OD_600_ was observed at 72 h in cultures grown at pH 6.5. Therefore, the optimum growth of AC157Xtp^T^ can be obtained at pH 6–7, with the best growth at pH 6.5 (Table S9). The combined information on the optimal growth conditions for AC157Xtp^T^ is presented in Table 3.

**Table 3.** Summary of the optimal growth conditions for strain AC157Xtp^T^

AC157Xtp^T^ strain	Agar media	Broth	Temperature	Salinity	pH	Oxygen
Tolerated	CBAb, TSAb, HIAb, MHAb, 2X YTGA and HIA	CB, TSB, 2XYTG, Insectagro® DS2 and SFM4Insect™	25–40°C	0–1.5% w/v	5–7.5	5% CO_2_
Recommended	CBAb, TSAb, HIAb, MHAb	CB, TSB, 2XYTG, Insectagro DS2 and SFM4Insect™	30–35°C	0–0.5% w/v	6–7 (best at 6.5)	Anaerobic

### Cell/colony morphology

The cell morphology was determined by scanning electron microscopy (SEM). Briefly, 48-h-old colonies grown in HIAb medium were transferred to Aclar discs by touching single colonies (‘colony touch method’) and fixed overnight at room temperature with a fixative solution of 2.5% glutaraldehyde in 0.1 M sodium cacodylate buffer. Alternatively, colonies were submerged in the fixative solution overnight at room temperature in the Petri dishes (‘immersive method’) and transferred to microcentrifuge tubes. The third method consisted of transferring a droplet of 48-h-old SFM4Insect™ cultures to Aclar discs and adding the fixative solution after the complete drying of the droplet (‘liquid method’). Then, the fixative solution was removed, and samples were washed twice with 0.1M sodium cacodylate buffer. Subsequently, samples were subjected to 1 h of staining with 1% osmium tetroxide (OsO_4_) in 0.1 M sodium cacodylate buffer, washing with ultrapure water, 5-min cross-linking with 1% thiocarbohydrazide in ultrapure water, another wash with ultrapure water, 5-min staining with 1% osmium tetroxide (OsO_4_) in 0.1 M sodium cacodylate buffer and two final rounds of washing with ultrapure water. Samples were immersed in ultrapure water overnight at room temperature. Then, samples were dehydrated for 10–15 min with increasing ethanol concentrations (15%, 30%, 50%, 70%, 90% and 96% v/v), and two final rounds of 30 min dehydration with absolute ethanol (100% v/v) were performed. Finally, samples were subjected to chemical drying for 5–15 min with a 1:1 solution of absolute ethanol and hexamethyldisilazane (HMDS), followed by a 5–15-min drying step in 100% HMDS. The HMDS was removed, and samples were dried in a fume hood. Dry samples were then sputter coated (Cressington 208 Benchtop Sputter Coater) with a 5 nm platinum-palladium (Pt/Pd) layer. SEM images were obtained using a Zeiss Supra 40VP scanning electron microscope at 5 kV and a working distance of 8–12 mm.

**Fig. 5.**
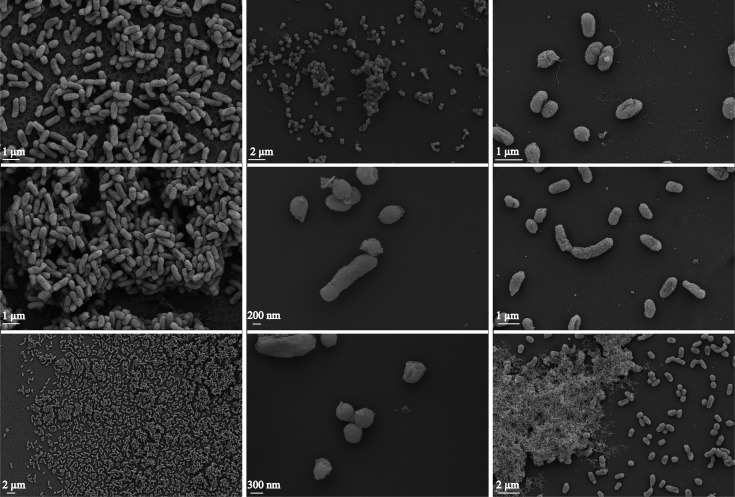
Scanning electron micrographs of cells of AC157Xtp^T^. The left, central and right columns represent samples prepared by the ‘colony touch’, ‘immersive’ and ‘liquid’ methods, respectively.

The predominant AC157Xtp^T^ strain morphologies observed using SEM consisted of coccoid and small rod-shaped cells. Measurements of at least ten different cells of the ‘colony touch’, ‘immersive’ and ‘liquid’ methods revealed sizes ranging from ~0.5 to 1.6 µm long and ~0.4 to 0.7 µm wide. No flagella were observed, and filamentous cells, reported for other *Orbaceae* [65,66], were also not observed (Fig. 5). Colonies growing in HIAb at 35°C for 48 h under anaerobic conditions have a ‘bubble-like’ appearance, being translucent, smooth, round and with entire margins. Five- to seven-day-old colonies present translucent margins and a whitish centre, resembling a ‘fried egg’ or, as described for *Orbus hercynius* [65], a ‘bulls-eye’ appearance.

### Biochemical/metabolic features

The AC157Xtp^T^ strain was also characterized for its biochemical and metabolic features. Following inoculation of a 48-h-old colony grown on HIAb into triple sugar iron (TSI) agar slants (Hardy Diagnostics), we observed that AC157Xtp^T^ is capable of fermenting glucose (dextrose), lactose and/or sucrose without gas or H_2_S production. We assessed the occurrence of cytochrome oxidase activity in the AC157Xtp^T^ strain by smearing 48-h-old cells growing in HIAb onto Oxistrips™ Oxidase Strips (Hardy Diagnostics), and a negative result was obtained. For determining catalase activity, 48-h-old isolated colonies growing in HIAb were collected with a sterile wooden stick and placed onto microscope slides. Then, a drop of 3% hydrogen peroxide (H_2_O_2_) was placed over the cells, and negative catalase activity (no bubbles) was observed (Table 4).

Further enzymatic activities were assessed by the Rapid ID 32A system (bioMérieux) by harvesting 48-h-old pure cells, adjusting the suspension to a 4.0 McFarland standard in 0.85% w/v saline and inoculating according to the manufacturer’s instructions with five replicates. The AC157Xtp^T^ strain was only consistently positive for *N*-acetyl-*β*-glucosaminidase. We observed variable results for three enzymes, which were negative in most replicates except for one positive replicate for arginine dihydrolase and two positives replicates for *β*-galactosidase 6-phosphate and *β*-glucosidase. Negative results were observed in all replicates for the remaining enzymatic activities including urease, *α*-galactosidase, *β*-galactosidase, *α*-glucosidase, *β*-glucosidase, *α*-arabinosidase, *β*-glucuronidase, mannose fermentation, raffinose fermentation, glutamic acid decarboxylase, *α*-fucosidase, nitrate reduction, indole production, alkaline phosphatase, arginine arylamidase, proline arylamidase, leucyl-glycine arylamidase, phenylalanine arylamidase, leucine arylamidase, pyroglutamic acid arylamidase, tyrosine arylamidase, alanine arylamidase, glycine arylamidase, histidine arylamidase, glutamyl-glutamic acid arylamidase and serine arylamidase (Tables 4 and S10).

Fatty acid methyl ester (FAME) analysis was performed by EMSL Analytical, Inc. (NJ, USA) to determine the cellular fatty acid composition of strain AC157Xtp^T^. Briefly, pure colonies of AC157Xtp^T^ had their fatty acids extracted using the MIDI, Inc. *Instant* FAME™ method from Sherlock® and run on a Hewlett Packard 6890 gas chromatograph. Fatty acids were identified by the MIDI Sherlock® Microbial Identification System version 6.0B using the Clinical Bacteria Library (IBA1). The most abundant fatty acids detected for the AC157Xtp^T^ strain were the summed feature 8 (C_18:1_* ω*7c and/or C_18:1_ *ω*6c) – 45.9%, followed by C_16:0_ (palmitic acid) – 35.0% and C_14:0_ (myristic acid) – 7.8% (Tables 4 and S11).

Overall, AC157Xtp^T^ exhibits a unique combination of traits: anaerobiosis (tolerates growth at 5% CO_2_), catalase-negative, oxidase-negative, and capable of fermenting glucose, lactose and/or sucrose without gas or H_2_S production. This profile resembles *F. perrara* PEB0191^T^, which is also anaerobic and oxidase-negative. However, PEB0191^T^ is catalase-positive. AC157Xtp^T^ clearly differs from *O. hercynius* CN3^T^, which is aerobic, oxidase-positive, and catalase-positive. The major fatty acids of AC157Xtp^T^ are summed feature 8 (C_18:1_ *ω*6c and/or C_18:1_ *ω*7c) at 45.9%, C_16:0_ at 35.0% and C_14:0_ at 7.8%. This profile is similar to that of *F. perrara* PEB0191^T^ (SF8=44.4 and C_16:0_=35.0), but not identical, since C_14:0_ is 5.2% in PEB0191^T^, and other fatty acids sum to 9.4%. These values differ from that of *O. hercynius* CN3^T^, especially because, in addition to SF8 (38.5%) and C_16:0_ (33.7%), CN3^T^ has higher abundances of SF2 (9.4%) and SF3 (10.7%) (Table 4).

**Table 4.** Summary of the main physiologic, morphological and biochemical features of AC157Xtp^T^ in comparison with reference *Orbaceae* strains

	AC157Xtp^T^ strain	*F. perrara* PEB0191^T^ [66]	*Gilliamella apicola* wkB1^T^ [67]	*Orbus hercynius* CN3^T^ [65]	*Z. entericus* IPMB12^T^ [68]
**Isolation source:**	Gut of a carpenter bee (*Xylocopa tabaniformis parkinsoniae*)	Gut of a honeybee (*A. mellifera*)	Gut of a honeybee (*A. mellifera*)	Faeces of wild boar	Gut of the superworm (*Zophobas morio*)
**Growth conditions:**					
Oxygen requirement	Anaerobic (tolerates growth at 5% CO_2_)	Anaerobic	Microaerobic (5% CO_2_), facultative anaerobic	Aerobic, facultative anaerobic	Facultatively anaerobic
Optimum temperature	30–35°C	37°C	37°C*	20–30°C	25–37°C
Optimum salinity	0–0.5%	x	x	x	0–2%
Optimum pH	6–7 (best at 6.5)	7	6.0–6.5	7.5	9–10
**Morphology:**					
Cell format	Coccoid to small rod-shaped cells	Rod-shaped cells	Rod-shaped cells/filamentous	Coccoid to rod-shaped cells/filamentous	Coccoid or rod-shaped
Cell size	~0.5–1.6 µm long × ~0.4–0.7 µm wide	2 µm long × 0.5 µm wide	1.5 µm long × 0.5 µm wide/>10 µm filaments	1–1.5×0.8 µm/>50 µm filaments	0.7–1.0 µm long × 0.5–0.6 µm wide
Colony morphology	‘Bubble-like appearance’, translucent, smooth, round, with entire edges	Smooth, round, flat, semi-translucent	Smooth, white, round colonies,	‘Bulls-eye’ appearance	Translucent, convex, round and smooth with entire edges
**Fermentation†**					
d-Glucose	+	+	−	+	+
d-Fructose	x	+	x	x	+
d-Mannose	−	+	x	x	+
d-Ribose	x	w	x	x	x
d-Arabinose	x	w	x	x	+
d-Galactose	x	−	x	x	+
d-Mannitol	x	−	x	x	+
d-Sorbitol	x	−	x	x	x
dl-Xylose	x	w	x	x	x
Sucrose	+*	w	x	x	+
Lactose	+*	−	x	x	x
Maltose	x	−	x	x	+
Melibiose	x	−	x	x	x
Raffinose	−	−	x	x	+
**TSI slant†**					
Gas production from fermentation	−	x	x	x	x
H2S production from fermentation	−	x	x	x	x
**Enzymatic activities†**					
Blood haemolysis	−	−	−	x	x
*β*-Galactosidase (PNPG)	−	−	+	−	−
*β*-Glucosidase (aesculin)	+/-	+	+	+	+
*α*-Arabinosidase (l-arabinose)	−	x	x	x	+
Catalase activity	−	+	−	+	−
Cytochrome oxidase activity	−	−	−	+	+
Urease (urea hydrolysis)	−	−	−	+	−
Nitrate reduction	−	−	−	+	+
Indole production	−	−	−	−	+
Gelatinase	x	−	−	−	−
Arginine dihydrolase	x	−	−	−	−
*N*-Acetyl-*β*-glucosaminidase	+	x	x	*x*	−
**Fatty acids (%):**					
C_10:0_	−	−	−	0.1	−
C_12:0_	0.3	−	−	0.2	7.00
C_14:0_	**7.8**	5.2	7.5	6.9	19.1
C_16:0_	**35.0**	35.0	31.7	33.7	19.3
C_17:0_	2.0	−	−	−	−
C_18:0_	2.2	3.3	1.3	0.4	1.5
C_16:1_ *ω*5c	0.1	−	−	0.2	−
C_18:1_ *ω*9c	−	0.8	−	−	−
SF2 (C_16:1_ isomer I and/or C_14:0_ 3-OH)	1.1	−	−	9.4	5.6
SF3 (C_16:1_ *ω*6c and/or C_16:1_ *ω*7c)	4.2	2.0	9.4	10.7	7.0
SF 5 (C_18:0_ anteisomer and/or C_18:2_ *ω*6,9c)	0.2	−	−	−	−
SF8 (C_18:1_ *ω*6c and/or C_18:1_ *ω*7c)	**45.9**	44.4	41.3	38.5	36.00
Others	1.2	9.4	8.8	0.10	4.50

†Symbols for metabolic activities: ‘+’ for presence/positive; ‘−’ for absence/negative; ‘x’ for not tested; ‘w’ for weak fermentation activity; ‘+*’ for positive for both or one or the other (TSI slant result).

*Growth was reported at 37 °C, but the optimum temperature was not experimentally determined.

## Taxonomic conclusion

We conclude that AC157Xtp^T^ represents a distinct lineage from all described *Orbaceae* genera and, therefore, is proposed as the type strain of a novel genus. Genomically, AC157Xtp^T^ shows ANIb, TETRA, dDDH and AAI values far below species thresholds and falls at the lower end of the *Orbaceae*-specific genus boundary range for POCP and AAI, supporting inter-generic divergence rather than affiliation to *Frischella* or *Orbus*, its closest relatives. Phylogenetically, both core-genome and single-copy orthologue trees recover AC157Xtp^T^ as a well-supported, independent lineage sister to – but not nested within – *Frischella* or *Orbus*, with branch lengths consistent with genus-level separation. The strain also displays a distinctive phenotype, combining anaerobiosis, catalase- and oxidase-negative reactions, fermentation profiles and a characteristic fatty acid composition. Ecologically, its association with the carpenter bee *Xylocopa tabaniformis parkinsoniae* contrasts with the hosts of *Frischella*, *Gilliamella*, *Orbus*, *Zophobihabitans*, *Utexia* and *Candidatus* Schmidhempelia bombi [66,72], reinforcing its independent evolutionary niche. Taken together, these results demonstrate that AC157Xtp^T^ does not fit within any existing genus in the family *Orbaceae* and strongly support its formal recognition as *Neffella* gen. nov.

## Description of *Neffella* gen. nov.

*Neffella* (Nef.fel'la. N.L. fem. n. *Neffella*, named in honour of Dr. John L. Neff, a naturalist specializing in the biology, ecology and taxonomy of bees native to TX, USA).

Cells are Gram-negative, with coccoid to small rod-shaped morphology. Metabolism is anaerobic, with positive glucose fermentation. Does not produce gas or H_2_S from glucose, sucrose and/or lactose fermentation. Negative for catalase, oxidase, urease, nitrate reductase, indole production and *β*-galactosidase, variable for *β*-glucosidase and positive for *N*-acetyl-*β*-glucosaminidase activities. Occurs in the gut of the large carpenter bees (*Xylocopa*). The type species is *Neffella xylocopae*, isolated from the gut of *Xylocopa tabaniformis parkinsoniae*. The G+C content of the type species is 36.57 mol%.

## Description of *Neffella xylocopae* sp. nov.

*Neffella xylocopae* (xy.lo.co'pae. N.L. gen. n. *xylocopae*, of the bee genus *Xylocopa*).

In addition to the characteristics of the genus *Neffella*, it presents the following properties . The coccoid to small rod-shaped cells can measure ~0.5–1.6 µm long and ~0.4–0.7 µm wide. Colonies have a ‘bubble-like’ appearance and are translucent, smooth, round and with entire margins. It grows well in CBAb, TSAb, HIAb and MHAb agar media and in CB, TSB, 2X YTG, Insectagro® DS2 and SFM4Insect™ liquid media. The optimum growth can be obtained anaerobically between 30 and 35 °C, 0–0.5% salinity and 6–7 (best at 6.5) pH. The most abundant fatty acids are summed feature 8 (C_18:1_* ω*7c and/or C_18:1_ *ω*6c), C_16:0_ (palmitic acid) and C_14:0_ (myristic acid). The type strain is AC157Xtp^T^ (=NCIMB 15593^T^=ATCC TSD-486^T^) isolated from the gut of the large carpenter bee *Xylocopa tabaniformis parkinsoniae* in Austin, TX, USA. The species epithet refers to its association with the gut of *Xylocopa* bees. GenBank accession number for the whole genome sequence of *Neffella xylocopae* AC157Xtp^T^ is CP133583, and for the 16S rRNA gene, it is PQ456091.

## Supplementary material

10.1099/ijsem.0.007119 Uncited Supplementary Material 1.

10.1099/ijsem.0.007119 Uncited Supplementary Material 2.

## Abbreviations

AAI: average amino acid identity
ANI: average nucleotide identity
ANIb: average nucleotide identity based on blast
ANIm: average nucleotide identity based on MUMmer
ASV: amplicon sequence variant
BHI: brain heart infusion
BIC: Bayesian information criterion
CB: Columbia broth
CBA: Columbia blood agar
CBAb: Columbia blood agar with 5% v/v sheep blood
CDS: coding DNA sequence
dDDH: digital DNA–DNA hybridization
DDH: DNA–DNA hybridization
FAB: fastidious anaerobic broth
FAME: fatty acid methyl ester
GGDC: Genome-to-Genome Distance Calculator
HIA: heart infusion agar
HIAb: heart infusion agar with 5% v/v sheep blood
HIB: heart infusion broth
HMDS: hexamethyldisilazane
LBA: lysogeny broth agar
MHA: Mueller-Hinton agar
MHAb: Mueller-Hinton agar with 5% v/v sheep blood
MRS: de Man, Rogosa and Sharpe (MRS)
ONT: Oxford Nanopore Technology
PGAP: Prokaryotic Genome Annotation Pipeline
POCP: percentage of conserved protein
RAST: Rapid Annotations using Subsystems Technology
SEM: scanning electron microscopy
TETRA: tetranucleotide-derived z-score correlations
TSA: trypticase soy agar
TSAb: trypticase soy agar with 5% v/v sheep blood
TSB: trypticase soy broth
TSI: triple sugar iron
2x YTG: 2X yeast extract tryptone broth + 0.3% w/v Dextrose/D-glucose
2X YTGA: 2X yeast extract tryptone agar medium with 0.3% w/v Dextrose/D-glucose added
