# Supplementary material for: Neffella xylocopae gen. nov., sp. nov., a novel host-specific gut symbiont of Xylocopa carpenter bees in the family Orbaceae
Source: Int J Syst Evol Microbiol. 2026 Jul 14;76(7):007119. doi: 10.1099/ijsem.0.007119 (PMC13372188; doi:10.1099/ijsem.0.007119)
Supplement: Uncited Supplementary Material 1. [file ijsem-76-07119-s001.pdf]

Supplementary Information

1. Bee collection, dissection, and gut homogenization

Twenty-three carpenter bees comprising the species *Xylocopa tabaniformis parkinsoniae* (Xtp), *Xylocopa micans* (Xm) and *Xylocopa virginica* (Xv) were collected using a net from September 27 to December 25, 2021, in several locations at Austin, Texas (Table S1). The bees were kept at 4 ºC to enable immobilization prior to the dissection, made using forceps and iris scissors cleaned with 70% ethanol, at the lab of Nancy Moran from the Department of Integrative Biology of The University of Texas at Austin. The dissected guts were homogenized in Insectagro® DS2 broth using sterile pestles, and the gut homogenates were stored in 40% glycerol at -80ºC for further applications.

2. Amplicon sequencing of bee gut homogenates

To direct the isolation of Orbaceae, 300 µL of each gut homogenate were subjected to genomic DNA extraction using cetyltrimethylammonium bromide (CTAB), with bead-beating, adapted from [1]. Extracted DNA was subjected to library preparation and amplicon sequencing of the V4 region of the 16S rDNA gene using the Illumina iSeq platform, with the Hyb515F_rRNA (TCGTCGGCAGCGTCAGATGTGTATAAGAGACAGGTGYCAGCMGCCGCGGTA) and Hyb806R_rRNA (GTCTCGTGGGCTCGGAGATGTGTATAAGAGACAGGGACTACHVGGGTWTCTAAT) primer pairs.

Raw data were processed using CUTADAPT and DADA2 [2] R package following the pipeline available at https://benjjneb.github.io/dada2/tutorial.html. In summary, we performed removal of adapters with CUTADAPT, then proceeded with filtering low-quality sequences, trimming, dereplication, denoising and chimera removal with DADA2. The amplicon sequence variants (ASV) table was generated from the forward reads only, and used in the naive Bayesian classifier method implemented in DADA2 for taxonomic classification of the ASVs using the trained version 138.1 of the SILVA database. Then, data were analyzed using "mctoolsr" (available at: https://github.com/leffj/mctoolsr), "phyloseq" [3], "ggplot2" [4] and "vegan" [5] in the R software. Mitochondria and chloroplast sequences were filtered from the dataset, and unclassified ASVs were submitted to phylogenetic analysis to obtain classification at least

at family level, as follows: BLASTn searches against NCBI nucleotide database were performed, and closely related sequences downloaded. Sequences were aligned using MAFFT [6], and the MEGA software [7] was used to determine the best nucleotide substitution model based on the Akaike information criterion (AIC) and generate maximum likelihood trees with a bootstrap of 1000 replications (trees not shown). The relative abundances of the ASVs were plotted in heatmaps to evaluate the microbial composition of the bees (Fig. S1). ASVs classified as *Orbaceae* formed a putative *Xylocopa*-specific clade with *Orbaceae* ASVs from a previous study [8] (Fig. S2).

Supplementary Figures

	Acetobacteraceae	Atopobiaceae	Bacillaceae	Bifidobacteriaceae	Enterobacteriaceae	Lactobacillaceae	Morganellaceae	Orbaceae	Pseudomonadaceae	Weeksellaceae	Other	NA
Xv_8	0	0	0	0	0	100	0	0	0	0	0	0
Xv_7	0	0	0	6.3	0	24.2	69.4	0	0	0	0	0
Xv_6	33.5	0	0	0	0	66.1	0	0.4	0	0	0	0
Xv_5	0	0	0	0	0	100	0	0	0	0	0	0
Xv_4	82.5	0	3.2	0	0	9.9	0	0	0	0	0	4.4
Xv_3	1.8	0	0	0	20.6	66.6	0	0	0	0	0	11.1
Xv_2	0	0	0	6.6	23.7	69.7	0	0	0	0	0	0
Xv_1	0	0	0	18.6	0	81.4	0	0	0	0	0	0
Xtp_9	34.9	0	0	10.7	0	12.8	9.6	14.6	12.8	4.5	0	0
Xtp_8	4.7	0	0	10.1	0	16.9	0	20.7	29.4	18.1	0	0
Xtp_7	0	0	0	0	0	12	14.2	8.4	57.2	8.2	0	0
Xtp_6	47.6	0	0	9	0	8.6	10.8	6.1	13.9	0	0	4.1
Xtp_5	0	0	0	11.3	0	12.3	27.8	13.2	21.8	13.6	0	0
Xtp_4	30.7	0	0	13.4	0	26.7	2.8	5.6	19.9	0	0	1
Xtp_3	0	2.7	0	20.6	0	46.2	0	0	16.9	13.6	0	0
Xtp_2	3.9	0	0	10.5	0	47.3	22.9	4	6.9	4.5	0	0
Xtp_1	27.4	0	0	10.5	0	4.9	15.1	11.1	25.6	3.6	0	1.9
Xm_6	0	0	0	0	0	32	5.5	7	55.5	0	0	0
Xm_5	0	0	0	15.2	0	25.3	43	0	0	14.6	0	2
Xm_4	6.6	0	0	12.7	0	20.3	2.5	33.6	0	24.3	0	0
Xm_3	0	0	0	0	0	70.1	0	7.8	16.6	5.4	0	0
Xm_2	0	0	0	3.4	0	38.9	47	10.7	0	0	0	0
Xm_1	0	0	0	9.4	0	21.1	13.4	9.7	46.5	0	0	0

Figure S1 - Heatmap representing the relative abundances of bacterial families in each bee collected. Rows represent gut homogenates from individual bees of *Xylocopa virginica* (Xv), *Xylocopa tabaniformis parkinsoniae* (Xtp), or *Xylocopa micans* (Xm). Columns represent the bacterial families detected in our dataset. Darker blue squares indicate higher relative abundances.

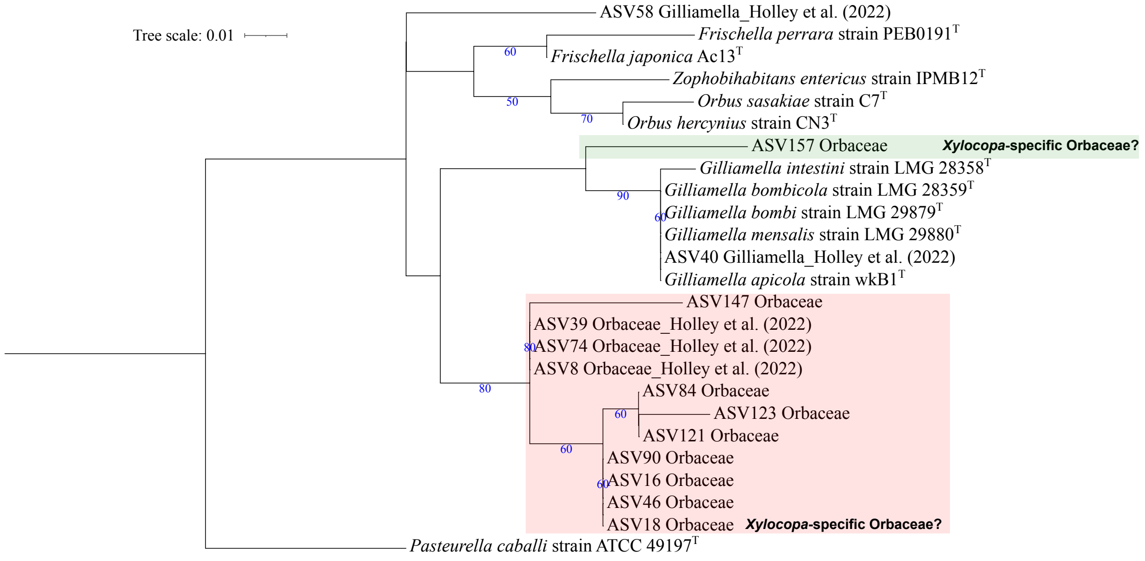

Figure S2 – Maximum-likelihood tree showing the placement of *Orbaceae* ASVs in relation to reference strains within the family *Orbaceae*. The tree was generated using MEGA, and the values on branches represent bootstrap percentages based on 1000 replicates. *Orbaceae* ASVs from *Xylocopa* were analyzed together with previously published *Orbaceae* ASVs [8] and 16S rRNA gene sequences from the NCBI RefSeq database.

References:

1. **Powell JE, Martinson VG, Urban-mead K, Moran A**. Routes of Acquisition of the Gut Microbiota of the Honey Bee Apis mellifera. *Appl Environ Microbiol* 2014;80:7378–7387.

2. **Callahan BJ, Mcmurdie PJ, Rosen MJ, Han AW, Johnson AJA, *et al.*** DADA2 : High-resolution sample inference from Illumina amplicon data. *Nat Methods* 2016;13:581–583.

3. **McMurdie PJ, Holmes S**. phyloseq : An R Package for Reproducible Interactive Analysis and Graphics of Microbiome Census Data. *PLoS One* 2013;8:e61217.

4. **Wickham H**. *ggplot2: Elegant Graphics for Data Analysis*. Springer-Verlag New York; 2016.

5. **Oksanen J, Blanchet FG, Friendly M, Kindt R, Legendre P, *et al.*** *vegan: Community Ecology Package*.

6. **Katoh K, Misawa K, Kuma KI, Miyata T**. MAFFT: A novel method for rapid multiple sequence alignment based on fast Fourier transform. *Nucleic Acids Res* 2002;30:3059–3066.

7. **Kumar S, Stecher G, Tamura K**. MEGA7: Molecular Evolutionary Genetics Analysis Version 7.0 for Bigger Datasets. *Mol Biol Evol* 2016;33:1870–1874.

8. **Holley JAC, Jackson MN, Pham AT, Hatcher SC, Moran NA**. Carpenter Bees (*Xylocopa*) Harbor a Distinctive Gut Microbiome Related to That of Honey Bees and Bumble Bees. *Appl Environ Microbiol*;88. Epub ahead of print 2022. DOI: 10.1128/aem.00203-22.
